# Pharmacokinetics of caspofungin acetate to guide optimal dosing in cats

**DOI:** 10.1371/journal.pone.0178783

**Published:** 2017-06-02

**Authors:** Jana Leshinsky, Andrew McLachlan, David J. R. Foster, Ross Norris, Vanessa R. Barrs

**Affiliations:** 1 Sydney School of Veterinary Science, Faculty of Science, The University of Sydney, Camperdown, New South Wales, Australia; 2 Faculty of Pharmacy and Education and Research on Ageing, The University of Sydney, Camperdown, New South Wales, Australia; 3 Concord Hospital, Concord, New South Wales, Australia; 4 School of Pharmacy and Medical Sciences, Australian Centre for Pharmacometrics, Sansom Institute for Health Research, University of South Australia, Adelaide, South Australia, Australia; 5 Clinical Pharmacology Division, SydPath, St Vincent’s Hospital, Darlinghurst, New South Wales, Australia; 6 Clinical School, University of New South Wales, Darlinghurst, New South Wales, Australia; 7 School of Pharmacy, Griffith University, Gold Coast, Queensland, Australia; Colorado State University, UNITED STATES

## Abstract

Cats are the most common mammal to develop invasive fungal rhinosinusitis caused by cryptic species in *Aspergillus* section *Fumigati* that are resistant to azoles but susceptible to caspofungin. In this study nonlinear mixed-effects pharmacokinetic modeling and simulation was used to investigate caspofungin pharmacokinetics and explore dosing regimens in cats using caspofungin minimum effective concentrations (MECs). Plasma concentrations in healthy cats were determined using HPLC-MS/MS after administration of a single and seven consecutive daily intravenous doses of 1 mg/kg caspofungin. In the final pharmacokinetic model an optimum maximum concentration (C_max_): MEC ratio of 10–20 was used to guide caspofungin efficacy. Simulations were performed for dosing regimens (doses 0.25–2 mg/kg and 6–72 h dosing intervals) with and without inclusion of a loading dose. Using a 1 mg/kg dose C_max_ first dose was 14.8 μg/mL, C_max_ at steady state was 19.8 μg/mL, C_min_ was 5 μg/mL and C_max_: MEC was >20 in 42.6% of cats after multiple doses. An optimal C_max_: MEC ratio was achieved in caspofungin simulations using 0.75 mg/kg q 24 h or 1 mg/kg q 72h. However, at 1 mg/kg q 72h, C_min_ was < MEC (<1 μg/mL) in over 95% of the population. Using a loading dose of 1 mg/kg and a daily dose of 0.75 mg/kg thereafter, the Cmax: MEC was optimal and C_min_ was > 2.5 μg/mL for 98% of the population. Based on the modeling data this dosing regimen is likely to achieve target therapeutic concentrations, meet the proposed C_max_: MEC window and provide consistent exposure between doses.

## Introduction

Cats are the most common mammal to develop naturally occurring invasive fungal rhinosinusitis caused by cryptic species of *Aspergillus* in section *Fumigati* [1]. Sino-orbital aspergillosis occurs in immunocompetent cats, is usually fatal and is most frequently caused by two emerging pathogens in the *Aspergillus viridinutans* species complex, *A*. *felis* and *A*. *udagawae* [1]. These cryptic species in section *Fumigati* also cause invasive fungal infection (IFIs) in humans, usually in the form of invasive pulmonary aspergillosis [1–3].

In both humans and cats *A*. *udagawae* and *A*. *felis* cause infections with a protracted clinical course that spread across anatomical planes to involve contiguous tissues and are refractory to standard therapy [2, 4]. The minimum inhibitory concentrations (MICs) of azole and polyene antifungal agents against *Aspergillus viridinutans* complex species are higher than those for *A*. *fumigatus* [3]. Caspofungin is an approved echinocandin for treatment of human patients with invasive aspergillosis refractory to other antifungal therapy [5–12] and was used to cure a case of feline sino-orbital aspergillosis caused by *A*. *felis* that failed treatment with azoles and amphotericin B [4]. The pharmacokinetics of caspofungin have been evaluated in rodents, lagomorphs and primates [13, 14]. Among companion animals, the pharmacokinetics of micafungin, but not caspofungin have been determined in dogs and no echinocandins have been evaluated in felines [15].

The pharmacokinetics of caspofungin in cats cannot be extrapolated from other mammals studied thus far since cats have substantial differences in drug metabolism compared to other mammals, including deficiencies in multiple phase II metabolic pathways including N-acetyltransferase (NAT) [16]. Our objective was to determine the pharmacokinetics of caspofungin in healthy cats after an intravenous dose of 1 mg/kg and after 7 consecutive daily doses. This study used a modeling and simulation approach to explore caspofungin dosing regimens in cats designed to examine C_max_: MEC ratios after intravenous caspofungin infusions.

## Materials and methods

### Pharmacokinetic study

#### Animals

Eight healthy adult desexed domestic shorthair cats (4 females, 4 males) with mean age and body weight of 3.94 ± 1.84 years and 4.4 ± 0.56 kg, respectively were recruited for the study. The cats were healthy cats from a research colony owned by Eurofins SCEC Pty Ltd. (www.scec.net.au) and were returned at the completion of the study in good health. Results of a complete blood count, serum biochemical analyses and urine specific gravity performed 5 days prior to the start of the study were within reference intervals for all cats. Cats were housed individually and acclimatized to the clinical environment for three days prior to the start of the study with free access to food and water. The study was approved by the Animal Ethics Committee (AEC) of The University of Sydney (Approval no. 2015/775, 6^th^ March, 2015).

#### Drug administration and blood sampling

For blood sampling, a 20-guage 8 cm triple-lumen central venous catheter (MILACATH^®^, MILA International, Inc., Kentucky, USA) was inserted into the jugular vein using a modified Seldinger technique after sedation with IV medetomidine (10 μg/kg; Domitor^®^, Pfizer Animal Health Australia, West Ryde, NSW) and butorphanol (0.1 mg/kg; Ilium Butorgesic^®^, Troy Laboratories Pty Limited, Glendenning, NSW), 24 h prior to starting the study. For drug administration, a peripheral catheter (22G IV radiopaque catheter; OPTIVA^®^, Smiths Medical International Ltd, Lanchashire, UK) was placed into a cephalic vein. For the single dose study (n = 8 cats) caspofungin acetate (Cancidas^®^, Merck Sharp & Dohme (Australia) PTY Limited, South Granville, NSW) was administered by IV infusion (1 mg/kg), 0.2 mg/mL in sterile saline solution (0.9% sodium chloride, Abbott Laboratories, Abbott Park, IL) over 1 h. For the multiple consecutive daily dosing study (n = 6 cats, 3 male, 3 female) administration of caspofungin acetate (1 mg/kg IV infusion over 1 h) was repeated once daily (at the same time each day) for a further 6 days.

Blood (1 mL) was collected in lithium-heparin immediately before drug administration (time 0), and after IV administration at 0.5, 0.75, 1, 1.25, 1.5, 2, 3, 6, 9, 12, and 24 h. Blood samples were immediately centrifuged (12, 000 g for 5 min) and plasma was stored at -80°C until assay. For the multi-dose study, a single blood sample was collected before each daily infusion (48, 72, 96 and 120 h after the first dose) to ascertain steady state plasma concentrations and blood sampling was repeated at the same times as day 1 on day 7. Repeat biochemical analyses were performed on Day 8, 24 h after the final caspofungin dose and pharmacokinetic blood sample collection.

#### Analytical method

Caspofungin concentrations in cat plasma were determined using HPLC-MS/MS (tandem mass spectrometry). Blood samples were prepared for assay using protein precipitation by adding methanol (80 μL) to 20 μL of calibrators, controls (drug free) and unknown samples using a stable isotope of caspofungin as internal standard (in methanol). Analytical reference standard for caspofungin was supplied by Merck Sharp & Dohme (Whitehouse Station, New Jersey 08889, USA). Chromatography and analysis was performed on a Shimadzu UPLC coupled to an 8050 tandem mass spectrometer in positive ion ESI mode, monitoring the transition 547.6 > 538.5 and internal standard transition 549.5 > 86.2. A Waters BEH C_18_ column was used with gradient elution with mobile phase A (water with 0.1% formic acid) and mobile Phase B (acetonitrile containing 0.1% formic acid). The mobile phase gradient involved 100% A for 0.5 minutes and a linear gradient to 0% A by 4.5 minutes. After raising the gradient to 100% B at 4.5 minutes it was held there for 0.5 minutes before returning to initial conditions for a further minute, making a total run time of 6 minutes.

The assay was validated in accordance with the US FDA Guidelines for industry for bioanalytical method validation utilising pooled plasma from six untreated cats with known concentrations of caspofungin reference standard. Linearity was demonstrated over the caspofungin concentration range of 0.1 to 10.0 μg/mL. The assay accuracy and precision (4 replicates on 3 occasions of spiked caspofungin plasma samples at 0.2 and 9.0 μg/mL) showed total precision of 12.0 and 4.3% (expressed as co-efficient of variation), respectively. Accuracy was 103.4 and 96.7%, respectively. Carry-over was demonstrated to be no more than 1.0% of the lowest standard in blank matrix following injection of the highest standard (10.0 μg/mL).

### Population pharmacokinetics analysis and dose regimen simulations

#### Methods software

Pharmacokinetic model development employed nonlinear mixed-effects modeling using NONMEM version 7.3 [17], with the Wings for NONMEM 7.3interface (http://wfn.sourceforge.net) and IFort compiler. Modeling was performed using a Dell PowerEdge R910 server with 4 by 10 core Xeon 2.26-Ghz processors running Windows Server 2008 R2 Enterprise 64- bit software. Data manipulation and post-run processing of NONMEM output was conducted using the R data analysis language (Version 3.2.2) and the packages ggplot2, GGally, foreign, tidyr, Hmisc, gdata, doBy, plyr, grid, stringr with associated dependencies [17–28].

#### General modeling strategy

The base pharmacokinetic model was developed in a step-wise manner. Pharmacokinetic models were coded using the built-in ADVAN subroutines of NONMEM. Linear kinetic models with 1 and 2 compartments were evaluated. The First Order Conditional Estimation (FOCE) method was used to fit models. The base model was selected on the basis of mechanistic plausibility, visual inspection of goodness-of-fit diagnostic plots, the precision of parameter estimates (se% <30% for fixed, <50% for random effects parameters), and the lowest value of the Akaike’s information criterion (AIC; Eq 1) in accordance with the number of parameters and the final NONMEM derived objective function value (OBJ). The base model was also required to pass the covariance step.

AIC=OBJ+2*number of parameters (1)

Unless stated otherwise, population parameter variability (PPV) was represented using an exponential error model (Eq 2):

$$P_{j}=TVP*e^{\eta j}$$ (2)

where P_j_ is the individual value for the parameter in the j^th^ individual, TVP is the typical population value of P and ηj is an independent random variable with a mean of zero and variance ω^2^. PPV was systematically examined on each fixed-effect parameter. Models with and without covariance for P were investigated using the OMEGA BLOCK functionality of NONMEM.

A combined proportional (θ_prop_) and additive (θ_add_) residual unexplained error model of the caspofungin concentrations (C) was used, where estimation of a THETA was employed and epsilon was fixed to zero (Eq 3):

$$C_{ij}={\hat{C}}_{ij}+\sqrt{\theta_{prop}{}^{2}*{\hat{C}}_{ij}{}^{2}+\theta_{add}{}^{2}}$$ (3)

*C*_*ij*_ is the i^th^ concentration measured in the j^th^ individual, ${\hat{C}}_{ij}$ is the model predicted C_ij_, and θ_*prop*_ and θ_*add*_ are parameters representing the proportional and additive residual error, respectively. The influence of the additive component of the error was also examined by a comparison with a proportional only error model.

All base models were investigated with allometric scaling for total body weight (TBW) referenced to 4 kg and an exponent fixed of 0.75 for clearance parameters and 1 for volumes, although during base model development models was explored without allometric scaling on any parameter.

#### Covariate model building

The base model was screened for the influence of cat sex, guided by plots of individual parameter random effects versus sex if shrinkage was low. Sex was only retained in the model if there was a significant improvement in OBJ at the p <0.01 level.

#### Model evaluation

Visual Predictive Checks (VPC) were used to assess the appropriateness of the candidate base and final models, faceted for any included covariates as appropriate. For the VPC’s, the median, 5^th^ and 95^th^ percentiles of the prediction-corrected observations were compared against the empirical 95% confidence intervals (CI) of the median, 5^th^ and 95^th^ percentiles of caspofungin concentrations from 1000 simulations of the index (original) dataset. The predictive performance of the model was considered acceptable if the median, 5^th^ and 95^th^ percentiles of the prediction-corrected observed and simulated data agreed well.

#### Caspofungin dose regimen simulations

The final pharmacokinetic model for caspofungin in cats was used to perform dosing simulations designed to examine C_max_: minimum effective concentration (MEC) ratios following intravenous infusions of caspofungin. Simulations were performed utilising total drug in serum rather than unbound drug, as published data available for MEC values have only been reported for the total drug. Based on data from a murine model of invasive pulmonary aspergillosis an optimum C_max_: MEC ratio of 10–20 was chosen as a marker of caspofungin dose efficacy [29]. Simulations were performed for dosing regimens of 0.25 mg/kg at 6 h intervals; 0.5 mg/kg at 6 h, 12 h and 24 h dosing intervals; 0.75 mg/kg and 1 mg/kg at 24 h, 48 h and 72 h intervals; 2 mg/kg at 48 h and 72 h dosing intervals. Loading dose regimen simulations were also considered based upon the results of the constant-dose regimens. A MEC value of 1 μg/mL was chosen based on the epidemiological cut-off value (ECV) of caspofungin for wild type *A*. *fumigatus* sens. str [30] and on antifungal susceptibility MEC data from clinical human and feline isolates of *A*. *felis* [1] and *A*. *udagawae* [2, 31, 32]. The duration of infusion was adjusted to 1, 2 and 4 h in the 1 mg/kg and 2 mg/kg simulations. Concentrations were simulated at 0.1 h intervals for 1000 standard 4 kg cats for each dose regimen for a total of 7 days of dosing. The percentage of animals with a C_max_: MEC > 10, > 20 and 10–20 was calculated over the entire 7 days of dosing. Maximising the percentage of animals with a C_max_: MEC of 10–20 was the criteria for “goodness” of a regimen. Supplementary pharmacokinetic parameters were also derived from the model parameters: AUC_ss_ (determined from dose and clearance), distribution (t_1/2α_) and terminal (t_1/2β_) half-life (calculated from the individual values of the pharmacokinetic parameters for each animal), C_max_ (derived directly from the data as the concentration at the end of each infusion).

## Results

All cats completed the pharmacokinetic study and no changes were seen on complete blood counts and multiple biochemical analyses after consecutive daily dosing. Physical examination parameters were normal throughout the study except for minor transient hyperthermia in one cat (range 39.6–39.8°C, reference range 37.5–39.1°C) during the first caspofungin transfusion only and mild transient diarrhoea that resolved within 24 h of the final caspofungin infusion on Day 8 in another.

### Pharmacokinetic modeling

The two-compartment linear model provided the best (ΔOBJ >200) description of the plasma concentration-time profiles of caspofungin in cats compared to a 1-compartment pharmacokinetic model. The two-compartment base model included both proportional and additive residual error terms, and random effects on clearance (CL) and the central volume of distribution (V1). Clearance and V1 were highly correlated. This was best described using a scale factor for the PPV on V1 as a function of the PPV on CL (ΔOBJ > 100) in order to prevent very high shrinkage on V1. Inclusion of PPV on the peripheral volume of distribution (V2) and the inter-compartmental clearance (Q) had no effect on the OBJ (ΔOBJ > 0.1), gave very low estimates for PPV and resulted in failure of the covariance step. Removal of allometric scaling for weight resulted in a worsening of model fit (ΔOBJ > 6), and so was retained in the final model. No covariates demonstrated evidence of predictive performance, and none were included in the final model. The population pharmacokinetic parameter estimates of the final pharmacokinetic model for caspofungin in cats are presented in Table 1.

**Table 1:** Population pharmacokinetic parameters for caspofungin in cats.

Parameter (units)	Estimate	%RSE
**Structural**		
CL (mL/h) * (WT/4)^0.75^	17.5	7
V1 (mL) * (WT/4)	214	8.6
V2 (mL) * (WT/4)	143	8.3
Q (mL/h) * (WT/4)^0.75^	150	20
**Between subject variability (BSV) %CV (% shrinkage)**		
CL	18 (0)	21
V/F BSV scale factor	1.05 (na)	8.4
**Residual unexplained variability (RUV)**		
Proportional (%CV)	7.8	18
Additive (SD, μg/mL)	0.61	20

CL, clearance; V1, central volume of distribution; V2, peripheral volume of distribution; Q, inter-compartmental clearance; RSE, relative standard error.

All model parameters were estimated with very good precision (7–21% RSE). The inter-individual variability for clearance was low (18%) and had minimal shrinkage (< 1%). There was good agreement between the observed and predicted population as well as individually predicted concentrations, with no evidence of bias, consistent with unbiased plots of conditional weighted residuals versus concentration and time after dose.

**Fig 1:**
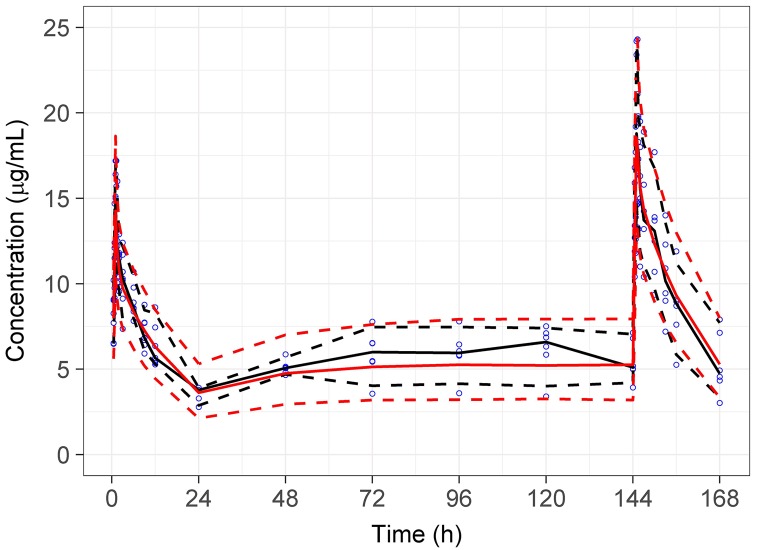
Visual predictive check plot for 1000 simulations of the final pharmacokinetic model and observed data. Solid lines are the median and dashed lines represent the upper and lower confidence intervals of the observed data (black) and prediction intervals of the final model (red). Circles are the raw observed data.

The visual predictive check based on 1000 simulations of the final PK model suggested acceptable fit of the model to the caspofungin plasma concentration-time data. Fig 1 shows the visual predictive check for simulations based on initial 24 h and final dose on day 7.

#### Caspofungin dose regimen simulations

Table 2 summarises the caspofungin model-independent pharmacokinetic parameters and exposure indices from the “best” simulations, as well as the regimen employed in the original study (1 mg/kg every q24 h). While increasing the infusion duration from 1 h to 2 h or 4 h had some impact on the observed C_max_, this has minimal impact of the different dose regimens in the ability to achieve target exposures. As a result, only the results from 1 h caspofungin infusions are presented here. Data for other dose regimens and infusion durations are presented in supporting material (S1 Table). Similarly, doses above 1mg/kg resulted in excessively high exposures, which were less able to meet the target C_max_:MEC ratio, and are not presented here. Data for other dose regimens and infusion durations are presented in supporting material (S1 Table).

**Table 2:** Summary of caspofungin model-independent pharmacokinetic parameters and exposure indices from simulations which result in the maximum % animals 10 < C_max_/MEC < 20 ratio for three dosing regimens administered as 1 h duration infusions (0.75 mg/kg q 24 h, 1 mg/kg q 24 h and 1 mg/kg q72 h).

Metric	Regimen	Mean	Median	SD	Minimum	Maximum
Distribution t_1/2_ (hr)		0.388	0.387	0.0287	0.280	0.481
Terminal t_1/2_ (hr)		14.5	14.5	0.963	12.0	19.4
AUC_ss,0-T_ (μg.h/mL)*	1 mg/kg	232	230	40.7	125	436
AUC_ss,0-T_ (μg.h/mL)	0.75 mg/kg	173	170	30.7	88.8	287
First dose						
C_max_ first dose (μg/mL)	1 mg/kg	14.8	15.0	2.11	8.55	23.8
C_max_ first dose (μg/mL)	0.75 mg/kg	11.0	10.8	1.61	6.01	16.6
AUC > MEC first dose (μg.h/mL)**	1 mg/kg q 24 h	133	133	22.0	69.0	227
AUC > MEC first dose (μg.h/mL)**	1mg/kg q72 h	163	159	36.4	75.9	313
AUC > MEC first dose (μg.h/mL)**	0.75mg/kg q 2 4h	93.2	92.5	16.6	42.7	148
Last dose						
C_max_ last dose (μg/mL)	1 mg/kg q 24 h	19.8	19.6	3.23	10.9	34.2
C_max_ last dose (μg/mL)	1 mg/kg q 72 h	15.4	15.3	2.38	9.27	23.8
C_max_ last dose (μg/mL)	0.75 mg/kg q24 h	14.7	14.2	2.42	7.94	22.9
AUC > MEC last dose (μg.h/mL)***	1 mg/kg q24 h	1350	1340	253	662	2560
AUC > MEC last dose (μg.h/mL)***	1 mg/kg q72 h	472	464	102	227	891
AUC > MEC last dose (μg.h/mL)***	0.75mg/kg q24 h	958	945	191	424	1650

* AUC_ss,0-T_ = the AUC within an inter-dosing interval (0-T), at steady-state, which is also equivalent to the AUC_0-infinity_ if only a single dose were given. This is identical for regimens that employ the same total dose (ie. the 1mg/kg q24h and 1mg/kg q72h).

**AUC > MEC first dose: this is the AUC > MIC for the first dosing interval. For the q24 regimens this is 24h. For the Q72h regimen this is 72h. The AUC > MEC in the first 24h for the q72h 1mg/kg regimen will be identical to that for the q24h 1mg/kg regimen as they both employ the same 1mg/kg dose.

***AUC > MEC last dose: this is the *cumulative* AUC>MIC during the entire 7 day dosing period.

In the typical 4 kg cat, a 1 mg/kg daily caspofungin dose results in a C_max_ of 14.8 μg/mL on the first dose while this is 19.8 μg/mL at steady-state, with trough concentrations of caspofungin approximately 5 μg/mL. In contrast, when given every 3 days, a 1 mg/kg dose reaches a mean C_max_ of 15.4 μg/mL and the average trough caspofungin concentrations are less than 1 μg/mL. A 0.75 mg/kg dose daily achieves a mean caspofungin C_max_ of 11.0 μg/mL after the first dose, 14.7 μg/mL at steady-state, with average trough concentrations of approximately 4 μg/mL.

**Fig 2:**
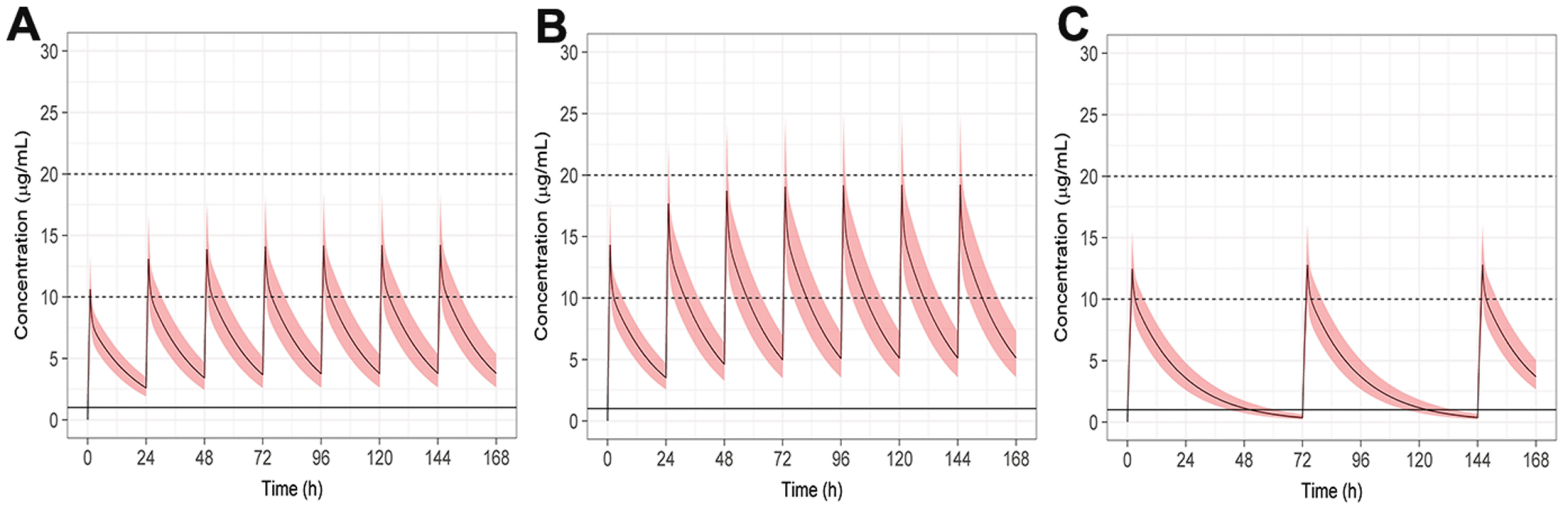
Plots of caspofungin concentration over time. A) 0.75mg/kg dosing daily for 7 days, B) 1mg/kg dosing daily for 7 days, C) 1mg/kg every 72 hours for 7 days. Solid black line indicates the median, shaded ribbon shows the 90% CI. Dashed black lines show the putative upper and lower C_max_ targets.

**Fig 3:**
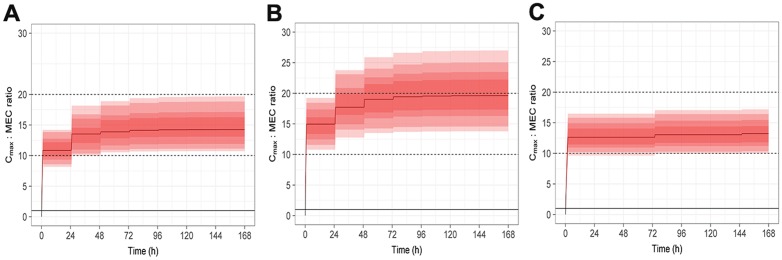
Plots of caspofungin C_max_:MEC ratio over time. A) 0.75mg/kg dosing daily for 7 days, B) 1mg/kg daily dosing for 7 days, C) 1mg/kg every 72 hours for 7 days. Red solid line indicates the median, shaded ribbons show the 95%, 90%, 75% and 50% CI’s in increasingly dark shading. Dashed black lines show the putative upper and lower C_max_:MEC ratio limits.

**Fig 4:**
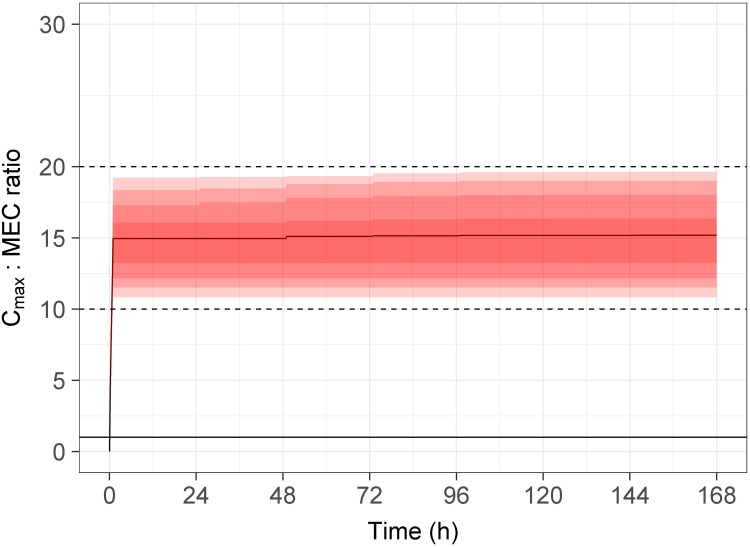
Plots of caspofungin C_max_:MEC ratio over time utilising a 1mg/kg loading dose on day 1 followed by 0.75 mg/kg daily thereafter. Red solid line indicates the median, shaded ribbons show the 95%, 90%, 75% and 50% CI’s in increasingly dark shading. Dashed black lines show the putative upper and lower C_max_:MEC ratio limits.

Plots of the median and 90% CI’s of the predicted caspofungin concentrations over time are provided in Fig 2, while Fig 3 shows the predicted distribution of the C_max_:MEC ratio over time. The maximal percentage of animals with a C_max_:MEC ratio of 10–20 occurred at 0.75 mg/kg once daily (q24 h) and 1 mg/kg every 3 days (q72 h). At the peak concentration on the first day of dosing with 0.75 mg/kg, 71% of animals were within the optimal C_max_: MEC, by the third and later daily doses this reached 97%. With 1 mg/kg dosing, > 96% of animals were within the target ratio after the first dose, and with continued q72 h dosing, this remained relatively constant for the remaining 7 days. Despite almost all animals being above the lower threshold, continued daily dosing with 1 mg/kg (the regimen employed in the original trial), resulted in fewer animals within the target window after subsequent doses, reducing to 76% on the second day, and 57% after the 7^th^ daily dose due to 43% of animals having a C_max_:MEC ratio greater than 20. A loading dose regimen, utilising 1mg/kg of caspofungin on day 1 followed by 0.75mg/kg q 24 h thereafter, resulted in 98% of cats falling within the optimal C_max_:MEC ratio from the first dose until the end of the study. Fig 4 shows the predicted distribution of the C_max_:MEC ratio over time for the loading dose regimen.

The differences between the regimens in terms of time-course of concentration relative to the MEC are highlighted in Table 2, demonstrating that the 1 mg/kg q72 h regimen results in a 50% lower AUC > MEC compared to the 0.75 mg/kg q24 h regimen.

## Discussion

This study has investigated the pharmacokinetics of caspofungin in healthy adult cats, finding that it is well tolerated and reaches therapeutic plasma levels at the dosing regimen studied. Using an innovative modeling and simulation approach we have shown that doses of 0.75 mg/kg or 1 mg/kg once daily, as well as 1 mg/kg every 72 hours, will reach target therapeutic levels. We have also shown that a loading dose of 1 mg/kg followed by 0.75mg/kg daily will enable 98% of cats to achieve the target therapeutic level after the first-dose.

Caspofungin exhibits linear pharmacokinetics in cats after intravenous administration of a single 1 mg/kg dose as demonstrated in other mammals including rodents (mice, rats), lagomorphs (rabbits) and primates (chimpanzees, rhesus monkeys and humans) [13, 14]. In humans, hepatic degradation of caspofungin to inactive metabolites occurs by non-enzymatic hydrolysis and N-acetylation [33–35]. Cats are deficient in NAT2 and possess a single NAT similar to human NAT1 but with lower affinity and activity [16]. Whether this is the major pathway of caspofungin metabolism in the cat is not known. The elimination half-life of caspofungin in cats (14.5 ± 0.96 h) was longer than that in healthy human adults administered an equivalent 70 mg dose (9.29 ± 1.96 h) [35].

Steady state was achieved at approximately 72 h with trough concentrations of caspofungin in cats at 1 mg/kg and 0.75 mg/kg daily of approximately 5 μg/mL and 4 μg/mL, respectively. In contrast, humans given 70 mg/kg of caspofungin daily reportedly only achieved steady state during the third week of administration with a lower trough concentration of 2.57 ± 0.34 μg/mL, as a result of a much longer terminal half-life in humans (40–50 h) compared to cats (15 h) [36]. The clearance we report in the typical 4 kg cat (18 mL/min) is larger on a per kg basis than that reported in the typical 70 kg human (10mL/min). We cannot comment on a comparison of volume of distribution as Stone et al [36] did not report this parameter, although it would appear that humans most likely have a larger volume of distribution per kg compared to cats.

Caspofungin was well tolerated by cats with only two adverse events (AE) observed—transient fever and diarrhoea. Since caspofungin exerts its fungicidal activity on the β-glucans, which are essential glucose polymer cell wall components of fungi but not mammals, AE in mammalian species are expected to be low compared to other antifungals [37]. AE in healthy humans administered caspofungin are minimal and mostly comprise infusion reactions [36]. Liver enzyme elevations did not occur in any cats in this study and were uncommon in healthy caspofungin-treated humans [36]. However, AE are higher in patients because of co-morbidities and altered drug metabolism. Rabbits in an IFI model had significantly slower clearance of anidulafungin associated with a higher plasma concentration of drug at the end of the dosing interval compared to healthy individuals [38]. For human patients with IFIs treated with caspofungin, hepatotoxicity and infusion reactions are most common AEs [39]. In caspofungin-treated patients with candidemia liver enzyme elevations (ALT, AST or ALP) were the most frequent laboratory abnormalities (13.9%) and the most common clinical AE were fever (7.5%), chills (5.3%), nausea or vomiting (5.3%) and phlebitis (3.5%) [39]. Other reported infusion reactions to echinocandins in humans, including rash, pruritus, facial swelling and anaphylaxis are mediated by histamine release [40].

Manifestations of acute anaphylaxis are species-dependent and directly related to the locations of the largest population of mast cells, which are the heart and lungs in humans, gastrointestinal tract and liver in dogs, and lungs in cats [41–48]. Thus, animal-models of disease need to account for such species differences and the effect of caspofungin in cats with IFIs needs to be evaluated in prospective studies.

Echinocandins have concentration-dependent activity against *Aspergillus* spp [49]. In this study, at a dose of 1 mg/kg q 24 h C_max_ and C_min_ were well in excess of the highest MECs reported for *Aspergillus viridinutans* complex species. Caspofungin had MECs of less than 0.06 μg/mL for clinical isolates of *A*. *felis* from cats with invasive aspergillosis, except for one isolate in which the MEC was 2 μg/mL [1]. However, other cryptic species in section *Fumigati* reported to cause invasive aspergillosis in humans and cats have shown decreased susceptibility to caspofungin, including some pathogenic isolates of *A*. *lentulus* which had MECs of caspofungin of 4 –>32 μg/mL [32].

In a murine model of invasive pulmonary aspergillosis caspofungin dose escalation and dose fractionation was evaluated and C_max_: MEC ratio was the parameter most strongly associated with reduced fungal burden, with an optimal value of 10–20. Mice dosed with 1 mg/kg q24 h or 2 mg/kg q48 h had a significantly lower fungal burdens than mice dosed with 1 mg/kg q6 h [29]. At higher doses of 4 mg/kg, correlating to a C_max_: MEC >20 there was a paradoxical effect on fungal growth resulting in increased burdens in mice. This paradoxical effect on fungal growth of *A*. *fumigatus* has also been observed *in vitro* for caspofungin [49], and both *in vitro* and *in vivo* for other first and second generation echinocandins [50, 51].

Although not proven in clinical trials, the paradoxical growth effect of caspofungin at C_max_: MEC > 20 in vivo has led to concerns about possible reduced clinical efficacy using dosing regimens where the C_max_ greatly exceeds the MEC [29]. In this study, using a dose regimen of 1 mg/kg q24 h, C_max_: MEC was > 20 in 42.6% of cats after multiple doses. Our simulated data shows that an optimal C_max_: MEC of 10–20 is achieved using 0.75 mg/kg q 24 h or 1 mg/kg q 3 days. However, at the higher dose with a 3 day inter-dosing interval, C_min_ is well below 1 μg/mL in more than 95% of the population as the prolonged dosing interval does not allow for drug accumulation. Using a daily dose of 0.75 mg/kg, the Cmax: MEC is below 20 and C_min_ is greater than 2.5 μg/mL for more than 95% of the population. This difference in exposure between doses is also highlighted by the 50% reduction in the cumulative AUC above the MEC for the 1 mg/kg q 72 h regimen compared to 0.75 mg/kg q 24 h. Although both 1 mg/kg q 72 h and 0.75 mg/kg q 24 h provide very similar results for C_max_: MEC, the latter regimen is vastly superior in regard to AUC > MEC. Thus, based on the modeling data, a daily dose of 0.75 mg/kg daily is likely to best meet the proposed C_max_: MEC window, as well as provide more consistent exposure between doses. Alternatively, utilising a loading dose regimen of 1mg/kg on day 1, followed by 0.75mg/kg daily will rapidly achieve steady state, as well as provide continual exposure between dosing.

The relationship between antifungal in vivo susceptibility, dosages, drug concentrations within various body compartments and toxicological effects are not completely understood. Although our pharmacokinetic analyses have attempted to predict a regimen appropriate for safe and effective use of caspofungin using in vitro MEC values, inherent biological factors can affect therapeutic outcome. For example, in sino-orbital aspergillosis systemic therapy may not be sufficient to penetrate the site of infection and reduce fungal burden. In addition, our work here is based upon total plasma concentrations, and not unbound drug, which may further confound the results. The echinocandins are highly protein bound, and this does not appear to vary across mammalian species investigated [14, 15, 33, 52]. The unique properties of caspofungin create a challenge in determining the precise level of protein binding [14]. However, studies have shown that caspofungin is bound to albumin in serum and that binding in mouse and human serum is equivalent, with an unbound fraction of caspofungin of 3–4%, which is concentration independent [14, 33]. Caspofungin has been shown to bind to albumin in serum [14, 33]. The plasma unbound fraction of caspofungin in the cat was not determined in this study, and although likely to be similar to that of humans and mice, based on similar albumin concentrations in these three species, this should be confirmed in future investigations. The current regimen employed clinically provides total plasma concentrations consistent with the C_max_:MEC ratio proposed in the present analyses, suggesting that our simulations are likely to be clinically relevant. Further pharmacodynamic studies are therefore required which can be linked with the pharmacokinetic data presented here, prior to a robust prospective clinical study to document clinical efficacy and safety of the proposed regimens in feline patients affected by sino-orbital aspergillosis.

## Supporting information

S1 Table Summary of caspofungin simulations. (RTF)
