# Supplementary material for: Pharmacokinetics of caspofungin acetate to guide optimal dosing in cats
Source: PLoS One. 2017 Jun 2;12(6):e0178783. doi: 10.1371/journal.pone.0178783 (PMC5456383; doi:10.1371/journal.pone.0178783)
Supplement: S1 Table — (RTF) [file pone.0178783.s001.rtf]

Summary of Caspofungin simulations
Each regimen has data provided for each dose of the 7 day regimen. This will be the 7th dose for the q 24h regimen and the 25th dose for the q 6h regimen.  However, for the q6h and q12h regimens only data for the last dose each day is provided. Note that for dose regimens, there are several infusion durations (1h, 2h and 4h).

Dose regimen	Period	Cmax/MIC	
Dose	Dose Interval (h)	Infusion Duration (h)	Time (h)	Dose Number	Ratio > 10 (%)	Ratio > 20 (%)	10 > Ratio > 20 (%)	
0.25mg/kg	q6	1h	1	First Dose	0	0	0	
0.25mg/kg	q6	1h	25	Fifth Dose	27.5	0	27.5	
0.25mg/kg	q6	1h	49	Ninth Dose	65.6	0	65.6	
0.25mg/kg	q6	1h	73	Thirteenth Dose	76.4	0	76.4	
0.25mg/kg	q6	1h	97	Seventeenth Dose	77.9	0	77.9	
0.25mg/kg	q6	1h	121	Twenty first Dose	78.5	0	78.5	
0.25mg/kg	q6	1h	145	Twenty fifth Dose	78.7	0	78.7	
0.5mg/kg	q12	1h	1	First Dose	2.40	0	2.40	
0.5mg/kg	q12	1h	25	Third Dose	88.6	0	88.6	
0.5mg/kg	q12	1h	49	Fifth Dose	95.5	0.600	94.9	
0.5mg/kg	q12	1h	73	Seventh Dose	97.5	2.00	95.5	
0.5mg/kg	q12	1h	97	Ninth Dose	97.7	2.60	95.1	
0.5mg/kg	q12	1h	121	Eleventh Dose	97.7	2.70	95.0	
0.5mg/kg	q12	1h	145	Thirteenth Dose	97.7	2.70	95.0	
0.5mg/kg	q24	1h	1	First Dose	2.10	0	2.10	
0.5mg/kg	q24	1h	25	Second Dose	25.2	0	25.2	
0.5mg/kg	q24	1h	49	Third Dose	44.3	0	44.3	
0.5mg/kg	q24	1h	73	Fourth Dose	44.3	0	44.3	
0.5mg/kg	q24	1h	97	Fifth Dose	44.3	0	44.3	
0.5mg/kg	q24	1h	121	Sixth Dose	44.3	0	44.3	
0.5mg/kg	q24	1h	145	Seventh Dose	44.3	0	44.3	
0.5mg/kg	q6	1h	1	First Dose	2.20	0	2.20	
0.5mg/kg	q6	1h	25	Fifth Dose	100	27.8	72.2	
0.5mg/kg	q6	1h	49	Ninth Dose	100	68.6	31.4	
0.5mg/kg	q6	1h	73	Thirteenth Dose	100	76.7	23.3	
0.5mg/kg	q6	1h	97	Seventeenth Dose	100	78.8	21.2	
0.5mg/kg	q6	1h	121	Twenty first Dose	100	79.5	20.5	
0.5mg/kg	q6	1h	145	Twenty fifth Dose	100	79.8	20.2	
0.75mg/kg	q24	1h	1	First Dose	70.9	0	70.9	
0.75mg/kg	q24	1h	25	Second Dose	97.4	0.300	97.1	
0.75mg/kg	q24	1h	49	Third Dose	98.4	1.60	96.8	
0.75mg/kg	q24	1h	73	Fourth Dose	98.4	1.60	96.8	
0.75mg/kg	q24	1h	97	Fifth Dose	98.5	1.70	96.8	
0.75mg/kg	q24	1h	121	Sixth Dose	98.5	1.80	96.7	
0.75mg/kg	q24	1h	145	Seventh Dose	98.5	1.90	96.6	
0.75mg/kg	q48	1h	1	First Dose	72.8	0	72.8	
0.75mg/kg	q48	1h	49	Second Dose	86.0	0	86.0	
0.75mg/kg	q48	1h	97	Third Dose	87.1	0	87.1	
0.75mg/kg	q48	1h	145	Third Dose	88.3	0	88.3	
0.75mg/kg	q72	1h	1	First Dose	72.6	0	72.6	
0.75mg/kg	q72	1h	73	Second Dose	79.0	0	79.0	
0.75mg/kg	q72	1h	145	Third Dose	80.9	0	80.9	
1mg/kg	q24	1h	1	First Dose	99.3	0.700	98.6	
1mg/kg	q24	1h	25	Second Dose	100	24.3	75.7	
1mg/kg	q24	1h	49	Third Dose	100	32.5	67.5	
1mg/kg	q24	1h	73	Fourth Dose	100	39.7	60.3	
1mg/kg	q24	1h	97	Fifth Dose	100	41.5	58.5	
1mg/kg	q24	1h	121	Sixth Dose	100	42.1	57.9	
1mg/kg	q24	1h	145	Seventh Dose	100	42.6	57.4	
1mg/kg	q24	2h	1	First Dose	1.60	0	1.60	
1mg/kg	q24	2h	25	Second Dose	96.1	0	96.1	
1mg/kg	q24	2h	49	Third Dose	99.8	6.50	93.3	
1mg/kg	q24	2h	73	Fourth Dose	100	14.6	85.4	
1mg/kg	q24	2h	97	Fifth Dose	100	17.7	82.3	
1mg/kg	q24	2h	121	Sixth Dose	100	19.3	80.7	
1mg/kg	q24	2h	145	Seventh Dose	100	20.0	80.0	
1mg/kg	q24	4h	1	First Dose	0	0	0	
1mg/kg	q24	4h	25	Second Dose	80.4	0	80.4	
1mg/kg	q24	4h	49	Third Dose	99.3	0.700	98.6	
1mg/kg	q24	4h	73	Fourth Dose	99.5	2.70	96.8	
1mg/kg	q24	4h	97	Fifth Dose	99.5	3.80	95.7	
1mg/kg	q24	4h	121	Sixth Dose	99.5	4.60	94.9	
1mg/kg	q24	4h	145	Seventh Dose	99.5	5.40	94.1	
1mg/kg	q48	1h	1	First Dose	99.8	2.10	97.7	
1mg/kg	q48	1h	49	Second Dose	99.9	5.70	94.2	
1mg/kg	q48	1h	97	Third Dose	99.9	5.70	94.2	
1mg/kg	q48	1h	145	Third Dose	99.9	5.80	94.1	
1mg/kg	q48	2h	1	First Dose	0.900	0	0.900	
1mg/kg	q48	2h	49	Second Dose	96.1	0	96.1	
1mg/kg	q48	2h	97	Third Dose	97.8	0.200	97.6	
1mg/kg	q48	2h	145	Third Dose	98.2	0.300	97.9	
1mg/kg	q48	4h	1	First Dose	0	0	0	
1mg/kg	q48	4h	49	Second Dose	79.2	0	79.2	
1mg/kg	q48	4h	97	Third Dose	90.1	0	90.1	
1mg/kg	q48	4h	145	Third Dose	91.4	0	91.4	
1mg/kg	q72	1h	1	First Dose	99.1	1.70	97.4	
1mg/kg	q72	1h	73	Second Dose	99.2	2.80	96.4	
1mg/kg	q72	1h	145	Third Dose	99.2	3.80	95.4	
1mg/kg	q72	2h	1	First Dose	2.20	0	2.20	
1mg/kg	q72	2h	73	Second Dose	96.4	0	96.4	
1mg/kg	q72	2h	145	Third Dose	97.2	0.300	96.9	
1mg/kg	q72	4h	1	First Dose	0	0	0	
1mg/kg	q72	4h	73	Second Dose	80.8	0	80.8	
1mg/kg	q72	4h	145	Third Dose	84.0	0	84.0	
2mg/kg	q48	1h	1	First Dose	100	99.6	0.400	
2mg/kg	q48	1h	49	Second Dose	100	99.7	0.300	
2mg/kg	q48	1h	97	Third Dose	100	99.7	0.300	
2mg/kg	q48	1h	145	Third Dose	100	99.7	0.300	
2mg/kg	q48	2h	1	First Dose	100	1.50	98.5	
2mg/kg	q48	2h	49	Second Dose	100	96.6	3.40	
2mg/kg	q48	2h	97	Third Dose	100	99.4	0.600	
2mg/kg	q48	2h	145	Third Dose	100	99.4	0.600	
2mg/kg	q48	4h	1	First Dose	2.20	0	2.20	
2mg/kg	q48	4h	49	Second Dose	100	76.7	23.3	
2mg/kg	q48	4h	97	Third Dose	100	90.1	9.90	
2mg/kg	q48	4h	145	Third Dose	100	91.0	9.00	
2mg/kg	q72	1h	1	First Dose	100	98.9	1.10	
2mg/kg	q72	1h	73	Second Dose	100	99.4	0.600	
2mg/kg	q72	1h	145	Third Dose	100	99.4	0.600	
2mg/kg	q72	2h	1	First Dose	99.0	1.00	98.0	
2mg/kg	q72	2h	73	Second Dose	100	94.7	5.30	
2mg/kg	q72	2h	145	Third Dose	100	95.9	4.10	
2mg/kg	q72	4h	1	First Dose	1.80	0	1.80	
2mg/kg	q72	4h	73	Second Dose	100	78.1	21.9	
2mg/kg	q72	4h	145	Third Dose	100	82.2	17.8	
